# Acute kidney injury after lung transplantation, incidence, risk factors, and effects: A Swedish nationwide study

**DOI:** 10.1111/aas.70014

**Published:** 2025-03-11

**Authors:** Edgars Grins, Johanna Wijk, Henrik Bjursten, Maria Zeaiter, Sandra Lindstedt, Göran Dellgren, Per Ederoth, Lukas Lannemyr

**Affiliations:** ^1^ Department of Anesthesiology and Intensive Care, Department of Clinical Sciences Lund University Lund Sweden; ^2^ Department of Cardiothoracic and Vascular Surgery, Anesthesia and Intensive Care Skane University Hospital Lund Sweden; ^3^ Department of Anesthesiology and Intensive Care Medicine, Institute of Clinical Sciences at the Sahlgrenska Academy, University of Gothenburg and Section for Cardiothoracic Anesthesia and Intensive Care Sahlgrenska University Hospital Gothenburg Sweden; ^4^ Lund Stem Cell Centre Lund University Lund Sweden; ^5^ Department of Molecular and Clinical Medicine Sahlgrenska Academy, Gothenburg University Gothenburg Sweden; ^6^ Department of Cardiothoracic Surgery Sahlgrenska University Hospital Gothenburg Sweden

**Keywords:** acute kidney injury, cardio‐pulmonary bypass, incidence, lung transplantation, perioperative risk factors

## Abstract

**Background:**

Acute kidney injury (AKI) is a serious complication after lung transplantation, but the reported incidence varies in the literature. No data on AKI have been published from the Swedish lung transplantation program.

**Methods:**

The aim of our study was to investigate the incidence, perioperative risk factors, and effects of early postoperative acute kidney injury (Kidney Disease Improving Global Outcomes [KDIGO] criteria) after lung transplantation. A retrospective, nationwide study of 568 lung‐transplanted patients in Sweden between 2011 and 2020 was performed.

**Results:**

The incidence of AKI (any grade) was 42%. Renal replacement therapy was used in 5% of the patients. Preoperative factors independently associated with increased incidence of AKI were higher body mass index (odds ratio [OR]: 1.07, 95% CI: 1.02, 1.12) longer time on transplantation waiting list (OR: 1.05 [1.01, 1.09]), re‐transplantation (OR: 2.24 [1.05, 4.80]) and moderate to severe tricuspid regurgitation (OR: 2.61 [1.36, 5.03]). Intraoperative factors independently associated with increased incidence of AKI were use of cardiopulmonary bypass (OR: 2.70 [1.57, 4.63]), increasing number of transfused red blood cell units, and use of immunosuppressive therapy other than routine (OR: 2,56 [1.47, 4.46]). A higher diuresis (OR: 0.70, 95% CI: 0.58–0.85) was associated with less incidence of acute kidney injury. Development of AKI was associated with increased time to extubation (median 30 h, IQR [9, 118] vs. 6 [3, 16]), length of stay in the intensive care unit (9 days [4, 25] vs. 3 [2, 5]) and increased rate of primary graft dysfunction (OR 2.33 [1.66, 3.29]) and 30‐day mortality (OR: 10.8 [3.0, 69]).

**Conclusions:**

Acute kidney injury is common after lung transplantation and affects clinical outcomes negatively. Preoperative factors may be used for risk assessment. The use of cardiopulmonary bypass is a potentially modifiable intraoperative risk factor.

**Editorial Comment:**

Acute kidney injury is a common complication after lung transplantation that severely influences patient outcomes. This large study of more than 500 patients treated over a decade identified potentially modifiable factors associated with the development of acute kidney injury.

## 1 INTRODUCTION

Lung transplantation (LTx) is increasingly used as a final treatment option in patients with end‐stage lung disease. Acute kidney injury (AKI) remains a frequent complication after LTx despite developments in surgical techniques, immunosuppression, and anesthesia management.^1^, ^2^, ^3^, ^4^

Several factors have been attributed to the development of AKI after LTx, such as pre‐operative kidney function, use of CNI, perioperative management, and the use of extracorporeal circulation.^5^, ^6^, ^7^ Renal ischemia is considered an important mechanism in the development of AKI, and the kidneys are vulnerable to hypoxia in the setting of major surgery and critical illness.^8^ Still, the mechanisms behind post‐transplant AKI are incompletely understood.

Postoperative AKI is associated with increased perioperative morbidity and mortality, chronic kidney disease (CKD), dialysis, and sometimes even kidney transplantation.^1^, ^2^, ^4^, ^9^, ^10^ Through “organ crosstalk” the bidirectional interaction between failing kidneys and other organs, AKI is increasingly recognized not only as an innocent bystander but rather as a driver of organ dysfunction.^11^ Concomitant alterations in fluid homeostasis, dysregulated inflammatory and immune responses, and oxidative stress affect the whole body. In addition, a postoperative impaired renal function is especially problematic in the lung‐transplanted population, considering the need for lifelong treatment with nephrotoxic substances, for example, calcineurin inhibitors (CNI). Changing perioperative practices and shifts in the selection of donor and recipient candidates may affect the generalizability of previously described outcome predictors.^12^ Therefore, identification of modifiable risk factors for AKI is important.

The objectives of this study were: (1) to investigate the incidence of early AKI in a nationwide cohort of all LTx recipients between 2011 and 2020, (2) to delineate the pre‐ and intraoperative risk factors associated with AKI, and (3) to examine the effects of AKI in the early post‐operative period.

## 2 METHODS

### 2.1 Study design

After approval from the Swedish Ethical Review Authority (Dnr 2020‐00423, Etikprövningsmyndigheten Box 2110, Uppsala), we performed a retrospective study of all lung recipients, single‐ or double‐lung transplantations in Sweden, between January 2011 and December 2020. Date of ethical approval 5 August, 2020. All transplantations were performed at either Skane University Hospital, Lund, or Sahlgrenska University Hospital, Gothenburg. The Ethical Committee waived the need for informed consent. Patients below 18 years of age, patients undergoing concomitant transplantation of additional organs (heart/liver), patients with preoperative renal replacement therapy, and patients who died within 48 h after the transplantation were excluded from the study. The study was performed and reported according to the STROBE guidelines.^13^

All patients were evaluated and accepted for LTx in accordance with the guidelines of the International Society of Heart and Lung Transplantation (ISHLT).^14^ The study data were obtained from medical records. Preoperative patient characteristics included the indication for surgery, comorbidities, underlying pulmonary diagnosis, and laboratory variables prior to surgery. Intraoperative data included the type of lung transplantation, surgical technique, urinary output, fluid balance, need for blood transfusions, and the use of cardio‐pulmonary bypass (CPB) or extracorporeal membrane oxygenation (ECMO). Postoperative data included serum creatinine (S‐Cr) (daily during the first 7 postoperative days), the number of blood transfusions within 24 h from the start of surgery, inotropic score on arrival at the intensive care unit (ICU),^15^ and the use of continuous renal replacement therapy (CRRT) within 7 days.

### 2.2 Patient management

Patients were anesthetized according to institutional clinical routine using fentanyl, propofol, and rocuronium. Patients without contraindications or planned use of extracorporeal circulatory support received a thoracic epidural catheter for per‐ and postoperative pain management. A double‐lumen endotracheal tube was used to enable single‐lung ventilation. The choice to use or not use extracorporeal circulatory support (CPB or ECMO) together with the choice of surgical incision (sternotomy, clamshell incision or thoracotomy) was made according to the operating surgeon and clinical routine of the transplanting site. Perioperative antibiotic prophylaxis consisted of either cefotaxime (or other cephalosporins), clindamycin (if allergy to penicillin), or imipenem. Patients with cystic fibrosis (CF) had a regimen directed at chronic bacterial colonization. The postoperative antibiotic regimen is further described in Supplement A.

### 2.3 Immunosuppression

All patients received induction therapy with intravenous methylprednisolone 0.5–1 g; the first half of the dose was administered after anesthesia induction, and the rest before reperfusion of the graft. Since 2016, no azathioprine was given preoperatively. Furthermore, all patients were given methylprednisolone, antithymocyte globulin (ATG), mycophenolate mofetil, and CNI (tacrolimus or cyclosporine) in a standardized way according to local practice. Detailed information on immunosuppression is given in Supplement A.

### 2.4 Postoperative care

The postoperative management of the patients was performed in a dedicated cardiothoracic ICU under the discretion of the attending anesthesiologists/intensive care physicians and according to institutional routine, in collaboration with lung transplant physicians and the surgeons.

### 2.5 Definitions and outcome

Our primary outcome was the incidence of AKI within 7 days from surgery, defined and classified according to the KDIGO serum creatinine criteria.^16^ Urinary output criteria were not used. Baseline serum creatinine was measured on the ward before the start of the surgery.

Secondary outcomes were pre‐ and intraoperative factors independently associated with AKI.

Preoperative CKD was defined as a glomerular filtration rate (GFR) < 60 mL/min/1.73 m^2^. Preoperative GFR was measured by iohexol or Cr‐EDTA clearance (measured GFR, mGFR) (98.1% of our patients) or estimated using the CKD Epidemiology Collaboration equation (CKD‐EPI) (estimated GFR, eGFR).^17^ Tricuspid regurgitation (TR) was assessed from preoperative echocardiography. The duration of anesthesia was defined as the time from the induction of anesthesia or from the start of insertion of the epidural catheter to the time when the patient left the operating room. Intraoperative diuresis was adjusted for weight and anesthesia time. Transfusions were recorded as the number of units of red blood cells (RBC) within the first 24 h after the start of surgery. Primary graft dysfunction (PGD) was defined according to the ISHLT criteria.^14^ Time to extubation was the time from arrival to the ICU. Time in the ICU is the time to first transfer from the ICU.

### 2.6 Statistical analysis

Descriptive statistics were calculated to describe the demographics of the cohort, where continuous variables were expressed as median and interquartile range [IQR] and categorical variables as frequency rates and percentages *n* (%).

Statistical analysis was initiated with a univariable logistic regression analysis with AKI as an outcome variable, and the significance level for inclusion in the multivariable analysis was set at *p* < 0.3. Separate analyses were performed for intraoperative and postoperative variables, and significant variables from the univariable analysis were introduced in a multivariable logistic regression model to identify variables associated with AKI. If strong co‐variability between variables was detected, variables with less clinical significance according to our evaluation were eliminated from the multivariable analysis. The level of statistical significance was set at 5%, and odds ratios (OR) with a 95% confidence interval (CI) were reported. When comparing postoperative outcomes for patients with AKI to those without AKI, the Mann–Whitney *U* test was used for numerical variables, and the chi‐squared test was used for categorical variables.

The analysis was performed using R 4.3.3 (R Core Team [2024]. R: A language and environment for statistical computing).

## 3 RESULTS

**FIGURE 1:**
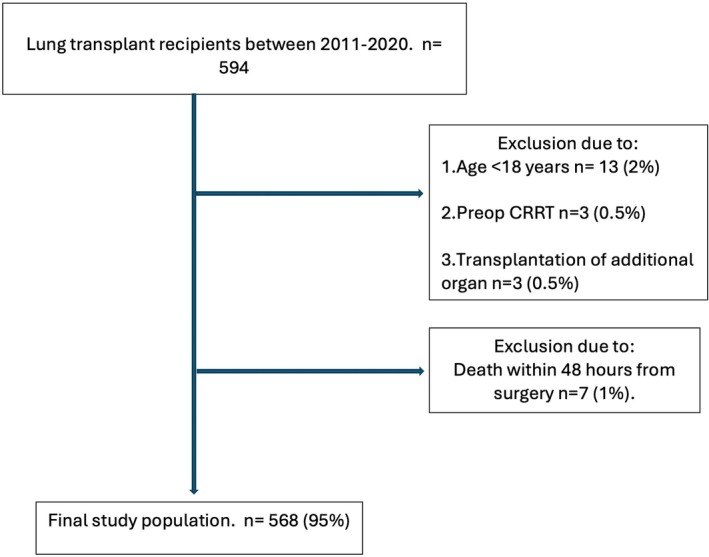
CONSORT diagram.

A total of 594 patients underwent LTx between 2011 and 2020 and were assessed for eligibility. Twenty‐six of these patients were found to be non‐eligible for reasons as shown in Figure 1.

### 3.1 Preoperative patient characteristics (Table 1)

**TABLE 1:** Baseline characteristics of lung transplant recipients with and without AKI.

Variables	All	No AKI	AKI
Total, *n*	568	327	241
Age, years	56 [48, 63]	57 [49, 63]	56 [47, 63]
Female gender	271 (48)	165 (50)	106 (44)
BMI, kg/m^2^	23.3 [20, 27]	23.2 [20, 26]	23.6 [21, 28]
Primary diagnosis, *n* (%)			
COPD	131 (23)	89 (27)	42 (17)
Idiopathic pulmonary fibrosis	152 (27)	84 (26)	68 (28)
Alfa‐1 at deficiency	58 (10)	34 (10)	24 (10)
Cystic fibrosis	62 (11)	31 (10)	31 (13)
Pulmonary hypertension	31 (6)	14 (4)	17 (7)
Other	134 (24)	75 (23)	59 (25)
Diabetes mellitus	81 (14)	39 (12)	42 (17)
Hypertension	99 (17)	54 (17)	45 (19)
CKD	52 (9)	22 (7)	30 (12)
Preoperative smoking	316 (56)	191 (58)	125 (52)
Re‐transplantation	58 (10)	26 (8)	32 (13)
Creatinine day of surgery, μmol/L	70 [58, 82]	68 [57, 80]	71 [59, 86]
mGFR waiting list, mL/min	84 [72, 96]	86 [74, 98]	78 [69, 92]
Day of surgery, mL/min eGFR	94 [80, 104]	95 [82, 105]	94 [78, 104]
ACE/ARB preoperative	63 (11)	33 (10)	30 (12)
Time on waiting list, months	1.9 [0.8, 5.2]	1.8 [0.8, 4.3]	2.2 [1.0, 6.6]
Systolic PA pressure, mmHg	36 [28, 55]	37 [30, 50]	35 [25, 55]
Tricuspid regurgitation			
No TR	311 (56)	195 (61)	116 (49)
TR grade 1	184 (33)	99 (31)	85 (36)
TR grade 2–3	62 (11)	25 (8)	37 (16)
Preoperative ECMO	21 (3.7)	8 (2.4)	13 (5.4)
Preoperative mechanical ventilation	31 (5.5)	11 (3.4)	20 (8.3)
Preoperative EVLP	23 (4.0)	11 (3.4)	12 (5.0)
Preoperative O_2_	341 (60)	190 (58)	151 (63)
Preop CNI	126 (22)	71 (22)	55 (23)
Ischemia time lungs, h	5.9 [4.4, 7.3]	6.0 [4.5, 7.3]	5.7 [4.4, 7.3]

*Note*: Values are presented as *n* (%) or median [IQR]. Other lung diagnosis includes CLAD (chronic lung allograft dysfunction), bronchiolitis obliterans, chronic pulmonary embolism, bronchiectasis, graft versus host disease of the lungs.

Abbreviations: ACE, angiotensin inhibitors; ARB, angiotensin blockers; BMI, body mass index; CKD, chronic kidney disease; CNI, calcineurin inhibitors; ECMO, extracorporeal membrane oxygenation; eGFR, estimated glomerular filtration rate (CKD‐EPI); EVLP, ex vivo lung perfusion; LTx, lung transplantation; mGFR, measured glomerular filtration rate.

In the whole patient cohort, the median age was 56 years, and 48% were women. Fourteen percent of the patients had diabetes mellitus, 17% had hypertension, and 56% of the patients had a history of smoking within 6 months prior to LTx. Median mGFR on the waiting list was 84 mL/1.73 m^2^ [72, 96] and 9% of the patients had CKD. Indications for LTx were chronic obstructive pulmonary disease (23%), idiopathic pulmonary fibrosis (27%), α1‐antitrypsin deficiency (10%), cystic fibrosis (CF) (11%), primary pulmonary hypertension (PPH) (6%), or other (24%). In total, 10% of the population were re‐transplantations. The median time on the waiting list was 1.9 months [0.8, 5.2]. At the time of LTx, 3.7% were on ECMO and (5.5%) were on mechanical ventilation. Mild TR, grade 1 was found in 33%, and moderate or severe, grade 2 or 3 in 11% and measured median systolic pulmonary arterial (PA) pressure was 36 mmHg [28, 55]. Patient characteristics for the whole cohort and across the groups with or without AKI are presented in Table 1.

### 3.2 Incidence of AKI

**FIGURE 2:**
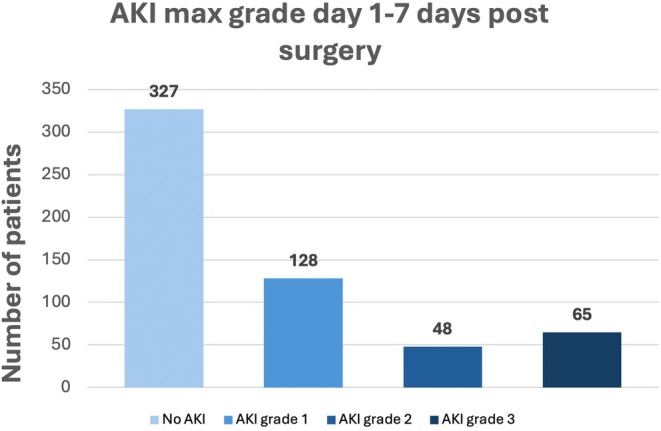
Bar chart diagram of the number of patients with different grades of AKI within the first 7 days after surgery.

**FIGURE 3:**
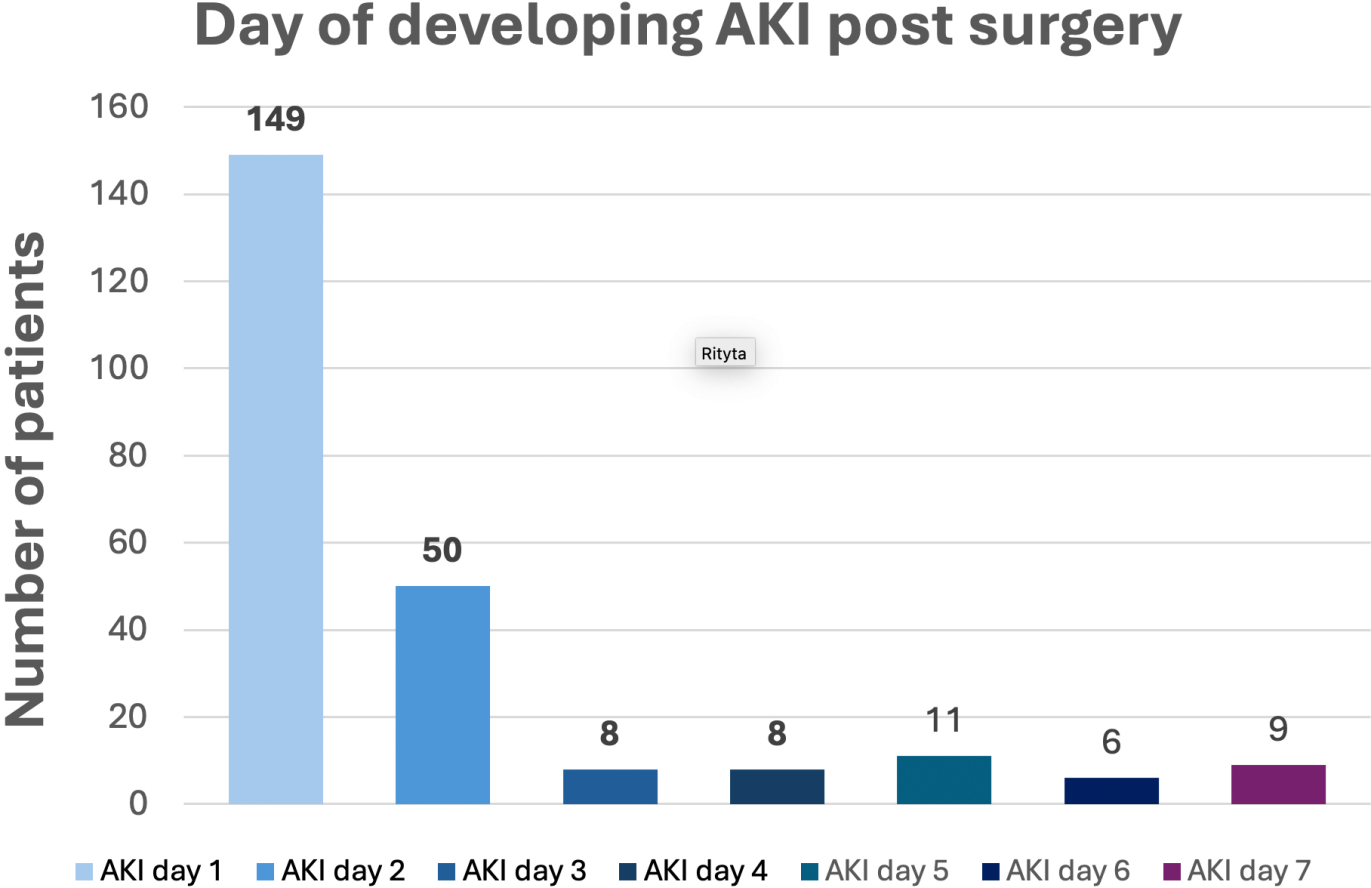
Bar chart diagram of the number of patients developing AKI stratified by day after surgery.

The incidence of AKI within 7 days from surgery was 42% (*n* = 241). Of these, 53% (*n* = 128) were grade 1, 20% (*n* = 48) grade 2, and 27% (*n* = 65) grade 3 (Figure 2). Renal replacement therapy was initiated within 7 days in 26 patients (5%) of the whole population (40% of patients with AKI grade 3). Most of the patients (82%) developed AKI within the first 2 days after surgery (Figure 3). No trend in AKI incidence over the study period was discovered.

### 3.3 Intraoperative variables (Table 2)

**TABLE 2:** Intraoperative variables of lung transplant recipients with and without AKI.

Variables	All	No AKI	AKI
Total, *n*	568	327	241
Type of LTx = DLTx	473 (83)	264 (81)	209 (87)
Type of incision			
Thoracotomy	339 (60)	238 (73)	101 (42)
Sternotomy	122 (22)	40 (12)	83 (34)
Clamshell	107 (19)	49 (15)	58 (24)
Intraop CPB = Yes	165 (29)	56 (17)	109 (45)
CPB duration	207 [174, 254]	180 [161, 229]	224 [186, 269]
Intraop ECMO = Yes	87 (15.3)	34 (10.4)	53 (22.0)
Intraop ECMO duration, min	297 [229, 431]	272 [230, 351]	357 [229, 480]
Surgery time, h	6.3 [5.2, 7.8]	6.2 [5.0, 7.4]	6.5 [5.3, 8.4]
Anesthesia time, h	9.3 [8.0, 11.1]	9.2 [7.8, 10.6]	9.6 [8.1, 11.4]
Intraoperative diuresis, mL/kg/h	1.1 [0.7, 1.9]	1.0 [0.7, 1.8]	1.2 [0.7, 2.0]
Nadir intraop hemoglobin, g/L	101 [86, 116]	105 [90, 119]	93 [82, 109]
Intraop bleeding			
≤600 mL	348 (63)	229 (72)	119 (51)
601–800 mL	38 (6.9)	18 (5.6)	20 (8.5)
801–1000 mL	43 (7.8)	23 (7.2)	20 (8.5)
1001–2000 mL	76 (13.7)	36 (11.3)	40 (17.0)
>2000 mL	49 (8.8)	13 (4.1)	36 (15.3)
Units of RBCs first 24 h			
0	224 (40)	178 (54)	46 (19)
1–2	109 (19)	67 (21)	42 (18)
3–6	118 (21)	49 (15)	69 (29)
7–12	69 (12)	23 (7)	46 (19)
>12	46 (8)	10 (3)	36 (15)
Intraop fluid, L	3.5 [2.0, 7.0]	2.7 [1.7, 4.7]	5.9 [2.7, 9.3]
Intraop fluid balance, L	1.4 [0.5, 3.1]	1.0 [0.4, 1.9]	2.4 [1.0, 4.3]
Other immunosuppression than ATG/Solumedrol	86 (15)	30 (9)	56 (23)
Antimicrobial prophylactic therapy			
Cefotaxime/other cephalosporin	328 (58)	212 (65)	116 (48)
Other	72 (13)	43 (13)	29 (12)
Carbapenem	167 (30)	72 (22)	95 (40)
Inotropic score	17 [10, 30]	16 [10, 25]	20 [10, 32]
Use of inhaled nitric oxide	94 (17)	54 (17)	40 (17)

*Note*: Values are presented as *n* (%); median [IQR] for all other variables.

Abbreviations: CPB, cardiopulmonary bypass; ECMO, extracorporeal membrane oxygenation; ATG, antithymocyte globulin.

Double‐LTx was performed in (83%) of all patients, and single‐LTx in 17%. Sternotomy was used in 22%, clamshell incision in 19%, and the remaining patients were operated on with uni‐ or bilateral sequential thoracotomies. Intraoperative CPB was used in 29% of the cases, with a median duration of 207 min [174, 254] and ECMO was used in 15%, with a median duration of 296 min [229, 431].

### 3.4 Preoperative variables associated with AKI (Table 3)

**TABLE 3:** Preoperative variables associated with acute kidney injury.

Variable	Univariable regression			Multivariable regression		
	OR	95% CI	*p*	OR	95% CI	*p*
Age	0.99	0.98–1.01	0.289	1.00	0.98–1.02	0.990
Gender (female)	0.77	0.56–1.08	0.127	0.77	0.53–1.14	0.190
Body mass index (kg m^2^)	1.05	1.01–1.09	0.009	1.07	1.02–1.12	0.002
Time on waiting list (month)	1.04	1.01–1.07	0.015	1.05	1.01–1.09	0.005
Re‐transplantation	1.77	1.03–3.06	0.040	2.24	1.05–4.80	0.037
Preoperative smoking	0.77	0.55–1.07	0.121	0.88	0.56–1.39	0.587
Diagnosis lung disease (ref: COPD)			0.081			0.488
Idiopathic pulmonary fibrosis	1.72	1.05–2.79	0.030	1.18	0.68–2.05	0.566
α 1‐Antitripsin deficiency	1.50	0.79–2.83	0.216	1.51	0.78–2.93	0.221
Cystic fibrosis	2.12	1.14–3.93	0.017	1.90	0.80–4.50	0.147
Primary pulmonary hypertension	2.57	1.16–5.71	0.020	1.10	0.40–3.01	0.853
Other	1.67	1.01–2.75	0.046	0.94	0.47–1.86	0.860
Diabetes	1.56	0.97–2.50	0.065	1.21	0.68–2.13	0.516
Hypertension	1.16	0.75–1.80	0.503			
CKD	1.97	1.11–3.51	0.021	1.43	0.69–2.96	0.334
eGFR day of surgery	1.55	0.80–3.01	0.191	1.00	0.46–2.17	0.994
Creatinine day of surgery	1.01	1.00–1.02	0.020			
Tricuspid regurgitation (ref: no TR)			0.003			0.008
TR grade 1	1.44	1.00–2.09	0.052	1.42	0.96–2.11	0.083
TR grade 2 and 3	2.49	1.43–4.34	0.001	2.61	1.36–5.03	0.004
Preoperative ECMO treatment	2.27	0.93–5.58	0.073	1.05	0.30–3.62	0.939
Preoperative mechanical ventilation	2.60	1.22–5.53	0.013	1.93	0.69–5.39	0.209
Preoperative ACE or ARB treatment	1.27	0.75–2.14	0.378			
EVLP	1.51	0.65–3.47	0.337			
Preoperative O_2_ treatment	1.21	0.86–1.70	0.274	1.09	0.76–1.58	0.638
Ischemia time lungs	0.99	0.92–1.06	0.692			
Preoperative CNI treatment	1.07	0.72–1.59	0.753			

*Note*: Other lung diagnosis includes CLAD (chronic lung allograft dysfunction), bronchiolitis obliterans, chronic pulmonary embolism, bronchiectasis, graft versus host disease of the lungs.

Abbreviations: ACE, angiotensin converting enzyme inhibitors; ARB, angiotensin receptor blocker; ECMO, extracorporeal membrane oxygenation; EVLP, ex vivo lung perfusion; TR, tricuspid regurgitation.

In the multivariable analysis, the following variables were independently associated with AKI: BMI (kg/m^2^) (OR: 1.07, 95% CI: [1.02, 1.12], *p* = 0.002), time on the transplantation waiting list (months) (OR: 1.05 [1.01, 1.09], *p* = 0.006), re‐transplantation (OR: 2.25 [1.05, 4.80], *p* = 0.038) and higher grade (2–3) of tricuspid regurgitation (OR: 2.61 [1.36, 5.03], *p* = 0.004).

### 3.5 Intraoperative variables associated with AKI (Table 4)

**TABLE 4:** Intraoperative variables associated with acute kidney injury.

Variable	Univariable regression			Multivariable regression		
	OR	95% CI	*p*	OR	95% CI	*p*
Type of surgery, double versus single	1.56	0.98–2.48	0.060	1.08	0.63–1.87	0.774
Type of incision (ref: Thoracotomy/bilateral sequential thoracotomy)			<0.001			
Sternotomy	4.83	3.10–7.53	<0.001			
Clamshell	2.79	1.79–4.36	<0.001			
Intraoperative extracorporeal circulation			<0.001			0.001
Cardiopulmonary bypass	5.43	3.61–8.15	<0.001	2.70	1.57–4.63	<0.001
ECMO	3.85	2.32–6.39	<0.001	1.41	0.74–2.70	0.301
Surgical time (h)	1.08	1.01–1.15	0.016	0.95	0.87–1.03	0.231
Anesthesia time (h)	1.08	1.01–1.14	0.019			
Diuresis/body weight/anesthesia time (mL/kg/h)	1.13	0.98–1.31	0.092	0.70	0.58–0.85	<0.001
Intraoperative bleeding (mL, ref: >600)			<0.001			
601–800	2.14	1.09–4.20	0.027			
801–1000	1.67	0.88–3.17	0.114			
1001–2000	2.14	1.29–3.53	0.003			
>2000	5.33	2.72–10.4	<0.001			
Units of red blood cells transfused during first 24 h (ref: 0 units)			<0.001			<0.001
1–2 units	2.43	1.47–4.01	<0.001	2.22	1.30–3.81	0.004
3–6 units	5.45	3.34–8.89	<0.001	4.10	2.26–7.42	<0.001
7–12 units	7.74	4.26–14.0	<0.001	5.91	2.76–12.6	<0.001
>12 units	13.93	6.44–30.2	<0.001	10.21	3.58–29.1	<0.001
HAES	0.84	0.43–1.64	0.613			
Intraoperative fluid balance (L)	1.26	1.16–1.36	<0.001	1.03	0.95–1.12	0.458
Other induction immunosuppression then ATG	3.00	1.85–4.84	<0.001	2.56	1.47–4.46	<0.001
Perioperative antibiotics			<0.001			
Cefotaxime/other cephalosporine (ref)	1.00		<0.001			
Other	1.23	0.73–2.08	0.43			
Carbapenem	2.41	1.65–3.53	<0.001			
Inotropic score	1.01	1.00–1.02	0.044	1.00	0.99–1.01	0.776
Use of inhaled nitric oxide	1.01	0.64–1.57	0.979			

Abbreviations: ATG, antithymocyte globulin; ECMO, extracorporeal membrane oxygenation; HAES, hydroxyethyl starch solution.

In the multivariable analysis, the following variables were independently associated with AKI: use of CPB (OR: 2.70 [1.57, 4.63], *p* < 0.001), intraoperative diuresis (mL/kg/h) (OR: 0.70 [0.58, 0.85], *p* < 0.001), increasing numbers of transfused RBC units (*p* < 0.001), and the use of induction immunosuppression other than ATG and methylprednisolone (OR: 2,56 [1.47, 4.46], *p* < 0.001). See Supplement A for details on immunosuppression.

### 3.6 Early outcomes and AKI

The 30‐day mortality in the whole transplanted cohort was 3%. In the non‐AKI group, 2/327 (0.6%) patients died within 30 days, while 15/241 (6.2%) patients died in the AKI group (OR: 10.8 [3.0, 69], *p* < 0.001). The median time to extubation in the non‐AKI versus the AKI group was 6 [3, 16] versus 30 h [9, 118] (*p* < 0.001), and the median ICU length of stay was 3 [2, 5] versus 9 [4, 25] days (*p* < 0.001). Primary graft dysfunction (any grade) occurred in 37% in the non‐AKI group versus 58% in the AKI group (OR 2.33 [1.66, 3.29], *p* < 0.001). Re‐operation because of bleeding occurred in 5.2% versus 20.3% (OR 4.65 [2.66, 8.53], *p* < 0.001) and of other causes than bleeding in 7.6% versus 19.1% (OR 2.85 [1.71, 4.85], *p* < 0.001) in the non‐AKI group versus the AKI group. There was no difference in the rate of re‐admission to the ICU between the groups; non‐AKI 7.6% versus AKI 12.0% (OR: 1.65 [0.94, 2.92]).

## 4 DISCUSSION

In this retrospective study, we present the incidence, pre‐ and intraoperative risk factors, and clinical effects associated with early AKI among all Swedish LTx recipients between 2011 and 2020. Among the 568 LTx patients, the total incidence of AKI after LTx was 42%, with most cases occurring within the first two postoperative days. The preoperative variables independently associated with AKI were a higher BMI, longer time on the waiting list, re‐transplantation, and the presence of moderate or severe tricuspid regurgitation. The intraoperative predictors of postoperative AKI were the use of CPB, low intraoperative diuresis, transfusion of erythrocytes, and the use of non‐standard immunosuppression. AKI was associated with significantly increased 30‐day mortality and worse early outcomes.

The incidence of AKI after LTx reported in the literature varies widely from 9 to almost 70%, depending on definition.^2^, ^3^, ^9^, ^18^, ^19^, ^20^ Moreover, the time at which AKI is diagnosed in different studies ranges from 72 h to 30 days postoperatively.^9^ After adjusting for different AKI definitions, a recent meta‐analysis showed a pooled AKI incidence of 52.5% and severe AKI requiring RRT of 9.3%.^4^ Although the incidence of AKI in our study is high (42%), it is still in the lower range as compared to earlier reports. Most patients were diagnosed with AKI on day 1 and day 2. In contrast, after coronary artery bypass (CABG) surgery, AKI is diagnosed first on day 2 and day 3.^7^ This suggests a more pronounced surgical trauma after LTx compared to CABG and strengthens the hypothesis that the trigger for renal injury mainly occurs during surgery.

The preoperative factors associated with AKI are rarely modifiable but may be of value in predicting the risk for postoperative AKI. In the present study, re‐transplantation and increasing time on the waiting list before transplantation may reflect a progressive multiorgan failure of long‐standing pulmonary disease. Previous studies have also identified higher BMI, time on the waiting list, and re‐transplantation to be predictors of post‐transplant AKI.^21^ Although higher BMI was associated with AKI, the numerical difference was very small.

To our knowledge, tricuspid regurgitation has not previously been associated with AKI after LTx. While a diagnosis of pulmonary artery hypertension is based on elevated pulmonary pressure, a tricuspid regurgitation may indicate a failing right ventricle. This condition is commonly accompanied by venous congestion and increased central venous pressure, which is strongly associated with AKI after cardiac surgery and in critically ill patients.^22^, ^23^, ^24^ Thus, a moderate to severe tricuspid regurgitation should be viewed as a potentially important risk factor for postoperative AKI.

In contrast to preoperative factors, the intraoperative variables in the study may to some extent be modifiable. The optimal use of mechanical circulatory support for LTx has been debated.^25^ It has been shown that extracorporeal circulation, and CPB in particular, induces several negative effects that may harm the kidneys. This may include increased bleeding in the anticoagulated patient and hemodynamic perturbances leading to impaired renal oxygenation, and the risk of AKI has been shown to increase with longer CPB duration.^22^, ^23^ Previous retrospective studies indicate that no mechanical circulatory support during LTx is superior to ECMO or CPB regarding renal outcomes.^26^, ^27^, ^28^ In the present study, both ECMO (OR 3.85, *p* < 0.001) and CPB (OR 5.43, *p* < 0.001) were associated with AKI in the univariable analysis, but only CPB remained significant in the multivariable analysis (OR 2.68, *p* < 0.001).

Among the intraoperative variables, transfusion of red blood cells and immunosuppression other than “standard” (ATG and methylprednisolone) were independently associated with postoperative AKI. Patients receiving non‐standard induction immunosuppressive therapy (described in more detail in Supplement A) may indicate a category with a high burden of donor‐specific antibodies and a high risk of immunological complications, and approximately one third in this group were re‐transplantations.^29^ Thus, other immunosuppression than standard may indicate more complicated patients deviating from the standard.^29^ Intraoperative transfusion of RBCs in LTx recipients has been linked to an increased risk of primary graft dysfunction (PGD) and mortality.^30^ In our study population, we found that transfusion of RBCs was associated with an incidence of AKI in a dose‐dependent manner, the highest risk in patients receiving more than 12 units of red blood cells (OR 10.2, *p* < 0.001). However, it is unknown whether this association is due to patient‐related factors, perioperative coagulopathy, adverse effects of blood transfusion, or the result of increased blood loss and hemodynamic instability in complex surgical procedures.^30^

In the present study, a lower intraoperative diuresis was a significant predictor of AKI. Previous studies have been conflicting regarding the link between oliguria and AKI in major surgery, and caution is advised in the interpretation.^31^ The cause‐and‐effect relation must be considered, as oliguria is a consequence of AKI. In addition, data on administered diuretics and the duration of low diuresis after surgery were not available for analysis in the present study. This finding should therefore be interpreted carefully.

The development of AKI as compared to non‐AKI was associated with increased morbidity. Furthermore, AKI was associated with increased duration of mechanical ventilation, increased ICU and hospital length of stay, number of reoperations both due to bleeding and other causes, as well as incidence of PGD. In addition, the 30‐day mortality was significantly increased in the AKI group as compared to non‐AKI. The challenging question remains whether efforts in reducing AKI after LTx could also have an impact on the outcomes described above.

### 4.1 Strengths and limitations

A strength of this study is that it is a large patient cohort and includes all consecutive patients who underwent LTx in Sweden during 2011–2020, accepted for LTx under similar conditions. Another strength is that we have no missing patients and very few missing values in the follow‐up of this national survey. We used the well‐defined KDIGO creatinine criteria, which make contemporary and future comparisons of AKI incidence between studies easier. A drawback is that a retrospective study precludes any conclusions regarding causality. Invasive measurement of the transpulmonary pressure gradient was not available for all patients, so the true number of pulmonary hypertension cases in our patients could not be determined. Lastly, we have not included any study variables concerning the donor, for example, age, gender, cause of death, or the matching between donor and recipient.

## 5 CONCLUSIONS

In this retrospective study, 42% of the LTx recipients developed AKI, the majority within the first two postoperative days, indicating a perioperative renal insult. Among the preoperative risk factors, tricuspid regurgitation suggests that cardiac function should be properly evaluated before listing for LTx. Intraoperative use—or avoidance—of mechanical circulatory support, particularly CPB, may be a modifiable risk factor for developing AKI. Other modifiable risk factors could be optimization of renal perfusion and diuresis in the perioperative period, choice of immunosuppression, and efforts to avoid bleeding and the need for transfusions. The described increased morbidity and mortality highlight the importance of a clinical determination to keep the incidence of AKI at a minimum.

## AUTHOR CONTRIBUTIONS

Study concept and design: EG, JW, LL, PE. Data acquisition: EG, JW, GD, SL, MZ. Scientific discussions and data analysis, all authors. All authors contributed to the critical revision and writing of the publication, and for the final approval to submit including accountability for the accuracy and integrity of the publication.

## FUNDING INFORMATION

This work was supported by the Swedish Heart and Lung Foundation (No. 20200260), the Swedish State under the government and the county councils, the ALF agreement (ALFGBG‐965102), Sahlgrenska University Hospital Funds (SU‐935106), Gothenburg Medical Society funds (GLS‐935113) and the Southern Health Care Region Research Funding. The statistical analysis was performed by statisticians affiliated with the framework Clinical Studies Sweden, a national collaboration supported by the Swedish Research Council.

## CONFLICT OF INTEREST STATEMENT

The authors declare no conflicts of interest.

## Supporting information


**Data S1:** Supporting Information.

## Data Availability

Research data are not shared.
