# Supplementary material for: Acute kidney injury after lung transplantation, incidence, risk factors, and effects: A Swedish nationwide study
Source: Acta Anaesthesiol Scand. 2025 Mar 11;69(4):e70014. doi: 10.1111/aas.70014 (PMC11894586; doi:10.1111/aas.70014)
Supplement: Supplementary file 1 — Data S1: Supporting Information. [file AAS-69-0-s001.docx]

# Supplement A

**Patient management**

***Immunosuppression protocol***

In general, the immunosuppressive protocol consisting of induction therapy followed by maintenance immunosuppression was used, however, the protocol has changed during the study period. Since 2016 the induction therapy consists of one single postoperative dose of rabbit anti-thymocyte globulin (ATG), instead of three and calcineurin-inhibitors are introduced already preoperatively.

Further, obvious differences between sites are described.

**Supplementary Table 1**: Differences in immunosuppressive therapy between sites and time periods.

| Site | Time period | Dose Intraoperative  methylprednisolone | Preoperative  Azathioprine | Induction therapy  ATG | Start CNI therapy |
| --- | --- | --- | --- | --- | --- |
| Site 1 | 2011 - 2020 | 1 g | 0 | 3 doses on day 1, 3 and 5 | Postoperatively  on day 1 - 5 |
| Site 1 | 2016 - 2020 | 1g | 0 | 1 dose day 1 | Preoperatively |
| Site 2 | 2011 – 2016 | 0,5 g | 2 mg/kg | 3 doses on day 1, 3 and 5 | Postoperatively on day 2 - 5 |
| Site 2 | 2016 - 2020 | 0,5 - 1 g | 0 | 1 dose day 1 | Preoperatively |

Methylprednisolone 0,5-1 g was administered before reperfusion of the allograft in all patients. Postoperative ATG (Thymoglobulin, Sanofi, Canada) 1-1,5 mg/kg was used in most of the patients, while highly immunized patients received basiliximab (Simulect, Novartis) as an anti-lymphocyte therapy. Most of the patients undergoing re-transplantation LTx were kept on maintenance therapy, and didn´t receive induction therapy.

Early postoperative maintenance immunosuppressive therapy consisted of corticosteroids (Prednisone 0,2 mg/kg/day), calcineurin-inhibitor (CNI) and purine synthesis inhibitor (azathioprine or mycophenolate mofetil).

Either tacrolimus or cyclosporine were used as a CNI. In early postoperative period the targeted blood concentrations for cyclosporine was 250 – 300 µg/L and 10-14 µg /L for tacrolimus. Patients receiving 3 doses of ATG were introduced to CNI on days 2-5 postoperatively, while patients receiving a single postoperative dose of ATG were introduced to CNI treatment preoperatively.

Azathioprine 1-2 mg/kg or mycophenolate mofetil 1 - 3 g/day was introduced on day 1 and dosages were adjusted to maintain white blood cell count over 4 x 10^9^/L. In recent years the mycophenolate mofetil has replaced azathioprine in the baseline regimen.

***Perioperative antimicrobial treatment***

According to standard for respective sites, either cefotaxime or imipenem was used as a

perioperative antimicrobial treatment. The antimicrobial regimen after that was individualized according to peri-operative tissue cultures.

Within 10 days postoperatively patients were commenced on a every other day trimethoprim-sulfamethoxazole prophylaxis for prevention of pneumocystis-jirovecii infection. Valganciclovir prophylaxis was used to prevent cytomegalovirus (CMV) reactivation in seropositive recipients or de novo infection in seronegative recipients. Actual post-organ transplantation CMV disease was treated with ganciclovir. Fluconazole was used as a general antifungal prophylaxis in the early postoperative period.
